# Design Method for Constant Force Components Based on Superelastic SMA

**DOI:** 10.3390/ma12182842

**Published:** 2019-09-04

**Authors:** Minghui Wang, Hongliu Yu, Ping Shi, Qiaoling Meng

**Affiliations:** 1 Shanghai Engineering Research Center of Assistive Devices/Institute of Rehabilitation Engineering and Technology, School of Medical Instrument and Food Engineering, University of Shanghai for Science and Technology, Shanghai 200093, China; 2 Department of Precision Mechanical Engineering, School of Mechatronic Engineering and Automation, Shanghai University, Shanghai 200444, China

**Keywords:** generalized design method, superelastic, shape memory alloy, constant force, finite element analysis

## Abstract

Clamping devices with constant force or pressure are desired in medical instruments, such as hemostatic forceps and the artificial sphincter, to prevent soft tissues from injures due to overloading. This paper studies the design method issues in constant force components using superelastic shape memory alloy. A generalized method for generating a constant force components-based shape memory alloy is proposed. An example of a C-shaped shape memory alloy sheet with a thickness of 0.2 mm is presented. The design results using the generalized design method for a C-shaped shape memory alloy sheet with 0.2 mm thickness are compared with its experimental results. Based on the generalized design method, the obtained design solutions for Cases 1 and 2 are coincident with the results obtained by the experiments. It could be seen that the generated design shape of the superelastic shape memory alloy component might obtain constant force within a relatively large deformation range. It is validated that the proposed generalized design method was feasible and effective. It is also illustrated that changing the geometric dimensions of the superelastic SMA component might obtain constant force within a relatively large deformation range.

## 1. Introduction

In medical instruments such as hemostatic forceps and the artificial sphincter, clamping devices for soft tissues of the human body are commonly used. It is considered that hemostatic forceps should satisfy at least three criteria, namely hemostasis, slip resistance, and atraumatic function. Although hemostatic forceps have been developed for over half a century, they continue to have the potential of exerting a harmful degree of pressure on blood vessels. In order to avoid soft tissue slippage and achieve hemostasis, excessive pressure is usually exerted, which may cause intimal lesions in blood vessels, sometimes resulting in the formation of thrombi that may induce ischemia and serious accidents [1,2,3]. The artificial sphincter also has same problem [4].

As overpressure can cause soft tissue injury and low pressure can cause soft tissue slippage, clamping soft tissues of the human body with constant force or pressure within a safe range is considered as an effective method to solve this issue. To realize the constant force property, different approaches can be adopted. Hydraulic, pneumatic, or electric drives are sometimes used. A control method for a pneumatic constant force tracking system was introduced [5]. A fractional-order proportional integral derivative (PID) control method was also introduced to achieve constant force hybrid control [6]. However, these systems usually need a complex control algorithm design, and they are more complicated than mechanical mechanisms [7]. Early constant force mechanisms were constant force tension springs, which are also known as Negator springs, which consist of a strip of spring steel in a tightly wound coil [8]. In many practical applications, constant force is generated by rigid components and linear springs. A rigid link constant force generator was also proposed [9]. A new curved surface constant force mechanism was designed, which mainly consists of a roller, curved surface, and the springs [10]. Alternatively, another way is to use compliant constant force mechanisms. A compliant constant force mechanism works on the basis of the deformation of its internal flexible members. A compliant constant force mechanism was developed so that it would be suitable for end-effector force regulation [11]. A strain energy storage device was provided for constant force behavior by the combination of a bending beam and cam [12,13]. However, these mechanisms increase the size and structure complexity and lacks compactness. A simple and compact structure is necessary for operation in hemostatic forceps, which provides limited space.

Here, it is shown that a constant force property can be realized by embedded superelastic shape memory alloys (SMA) in clamping devices to limit the clamping pressure. Shape memory alloys exhibit excellent biocompatibility and unique mechanical properties, which make them very attractive for highly miniaturized medical fields, such as orthopaedics, neurology, endoscopes, steerable catheters, prosthetic devices, cardiology, and interventional radiology [14,15]. The superelasticity of SMA is the ability to undergo large deformations in loading–unloading cycles without showing permanent deformations [16,17]. It is evaluated that the superelastic behavior of springs are made with the wire of shape memory alloys [18,19]. To obtain good constant force properties, the structure optimization of SMA is needed. In recent years, an optimized design of SMA was developed. An optimal design process for SMA wire-based micro-actuators was described and illustrated through a basic structure [20]. Design optimization of an SMA active needle for biomedical applications was also developed [21]. The structure optimization for a superelastic SMA component to obtain most constant force was done in previous studies by combining finite element analysis (FEA) in ANSYS with a genetic algorithm in MATLAB [22].

The purpose of the current work is to extend the past work [22], and we provide a generalized design method for generating constant force components-based superelastic SMA. Numerical simulation for SMA sheets of various shapes was done in previous studies, where it was found that varying the initial shape and geometric dimensions of a C-shaped SMA sheet may obtain approximately constant force in a relatively large deformation range. The C-shaped SMA sheets of 0.2 mm thickness are designed to obtain constant force in a large deformation range by using the proposed generalized design method. The design results are verified through comparison with experimental results of the prototypes.

## 2. Materials and Method

### 2.1. The Generalized Design Method for Generating Constant Force Components

**Figure 1:**
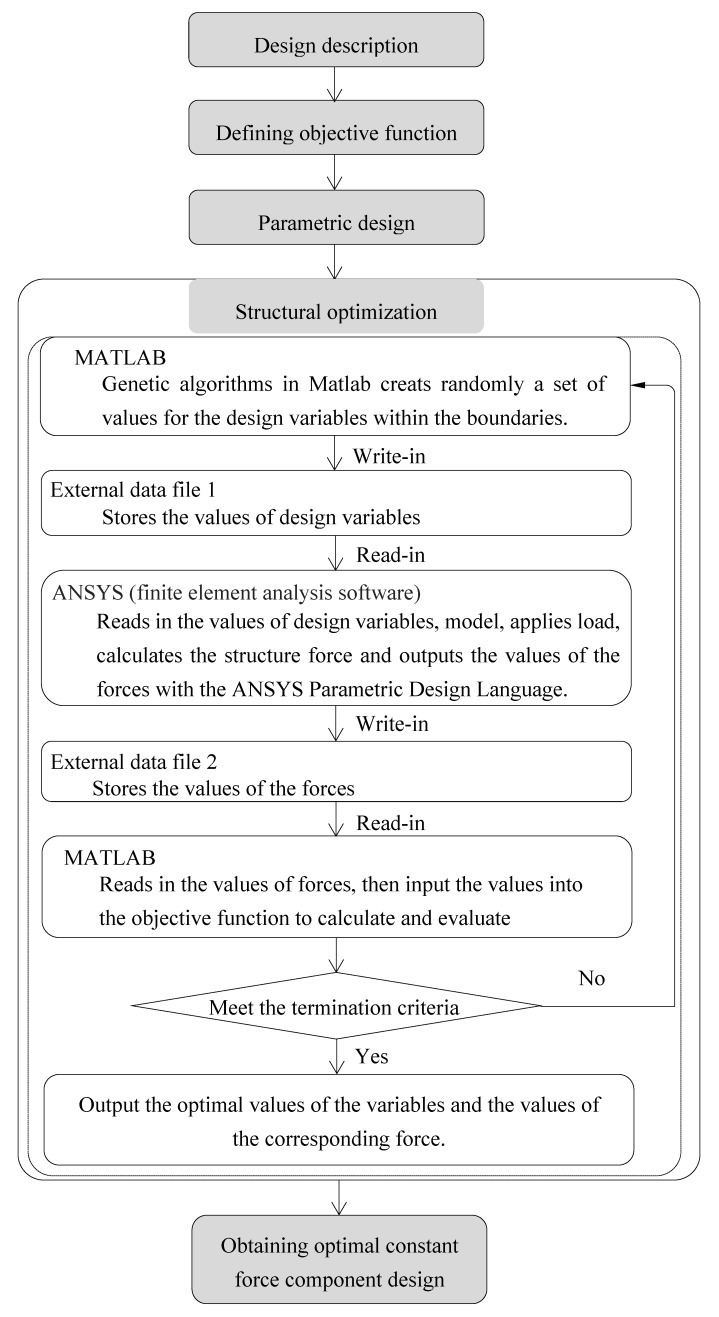
The generalized design method for generating constant force components.

The generalized design method described in this section entails design description, defining objective function, parametric design, and structural optimization. Figure 1 illustrates an overview of the generalized design method.

#### 2.1.1. Design Description

**Figure 2:**
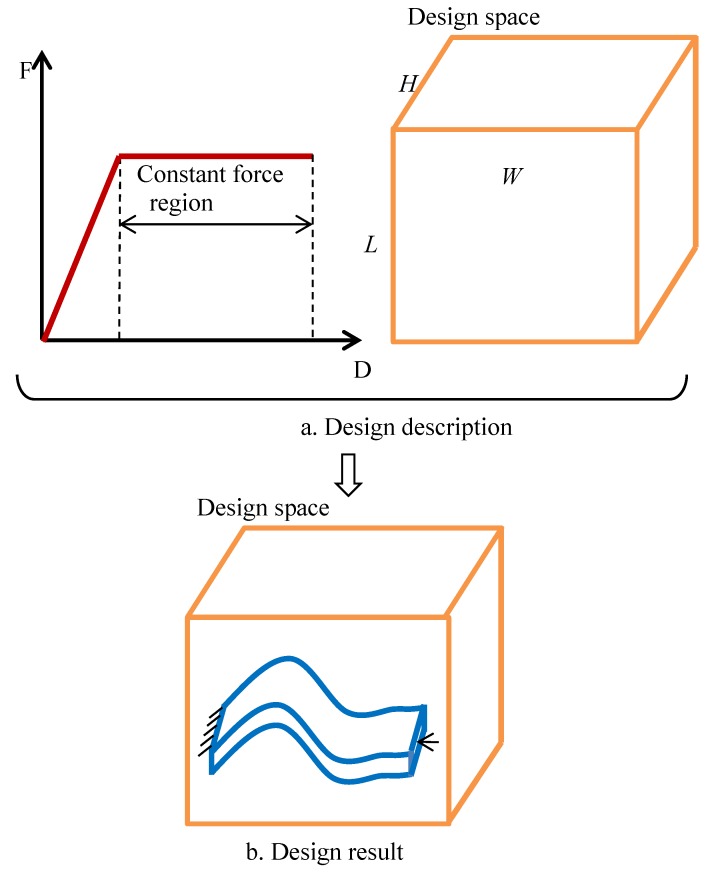
The illustration of the design problem. (**a**) Design description; (**b**) Design result.

The design description is defined by the following parameters: the prescribed load-displacement curve, dimensions of the design space, boundary conditions, and material property. The design space of the constant force element is length L, width W, height H, and unit mm, as shown in Figure 2.

#### 2.1.2. Defining Objective Function of Constant Force Component 

**Figure 3:**
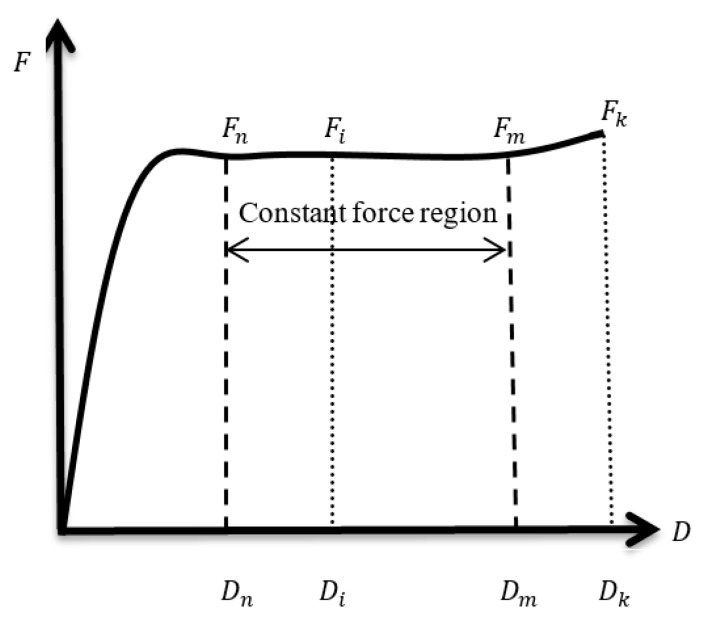
The force-to-displacement curve of a typical constant force component.

The force-to-displacement (*F−D*) curve of a typical constant force component is shown in Figure 3. The displacement of the *i*th point (range from 1 to *k*) is represented by *D_i_*, and the corresponding force of *D_i_* (the displacement at the *i*th point) is represented by *F_i_*. The forces of adjacent points are represented by *F_i_* and *F_i+_*_1_, respectively. The segment between *F_n_* and *F_m_* corresponding to displacement ranging from *D_n_* to *D_m_* is as the constant force region. The constraints with a force growth rate of two adjacent points less than 0.3% are defined to make the plateau stage as flat as possible. Obtaining the maximal displacements range that had a nearly constant force is defined as the optimization goal. Thus, the objective function could be formulated as:

The objective function = Max (*D_m_* − *D_n_)*/*D_k_* (1)

Subject to (*F_i+_*_1_ − *F_i_*)/*F_i_* < 0.3% (for *i* = *n* ~ *m*) (2)

#### 2.1.3. Parametric Design

To simplify the design, the work was limited that the initial shape of the SMA sheet was known. In order to obtain the best SMA structure with constant force characteristics, a parametric design of the model is needed.

#### 2.1.4. The Method of Structural Optimization

The structural analysis of superelastic SMA is a complicated nonlinear problem, due to its special mechanical properties. FEA was adopted to analyze the mechanical properties of superelastic SMA for its capability to analyze problems over complicated domains. ANSYS mechanical software, with its powerful computing capability, is a comprehensive FEA tool for structural analysis. Thus, ANSYS mechanical software is generally used to perform FEA of a superelastic SMA sheet. The superelastic material properties, rather than the shape memory model, were specified as the ANSYS material model, which were used to define the SMA component. The constitutive model on which this numerical method is based is called the Auricchio model [17]. The genetic algorithm is an optimization algorithm which utilizes natural selection and genetics with multiple initial designs, so it may explore a design space more thoroughly compared to other optimization algorithms. The genetic algorithm is a suitable optimization algorithm for superelastic SMA, which has non-smooth and nonlinear solution spaces. Accordingly, the genetic algorithm implemented in MATLAB was chosen as the optimization algorithm of the superelastic SMA sheet. The combination of finite element analysis with the ANSYS and genetic algorithm in MATLAB was applied to achieve the optimization design of the C-shaped SMA sheet. ANSYS is a subroutine that is called so by MATLAB. The objective function, the boundaries of design variables, and genetic algorithm are coded in the MATLAB program. Modeling, applying load, calculating the structure force, and outputting the values of the forces are coded in ANSYS parametric design language (APDL). Each design generated within the optimization routine’s genetic algorithm is evaluated using ANSYS [22].

### 2.2. An Example for Generating Constant Force Components Using the Generalized Design Method

**Figure 4:**
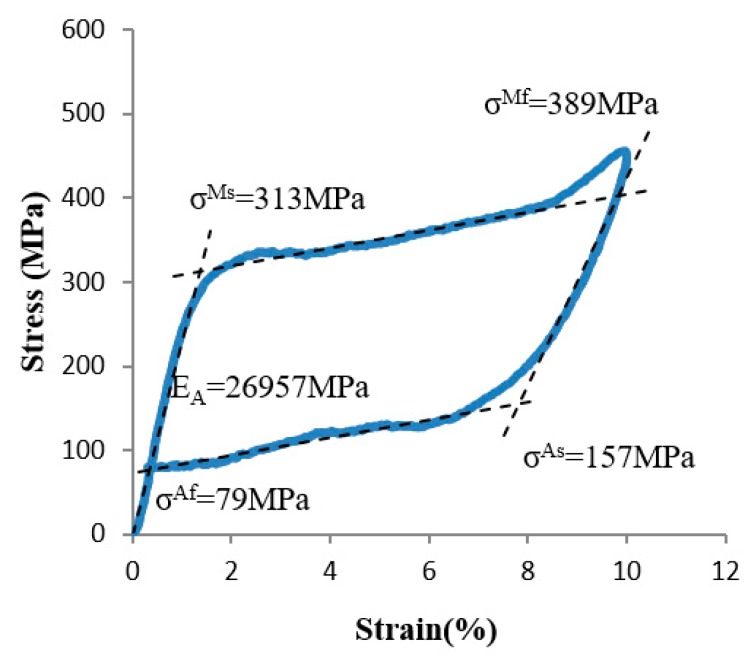
Tensile test curve of SMA sheet with a thickness of 0.2 mm.

A Ti-55.82at%Ni SMA was used as the material of the sheet with 0.2 mm thickness. The tensile test with an SMA sheet of 0.2 mm thickness is shown in Figure 4. The mechanical properties of the SMA are set as follows: from austenite to martensite, the stresses of the start and finish points of phase transformation are 313 MPa and 389 MPa, respectively, and from martensite to austenite, the stresses of the start and finish points of phase transformation are 157 MPa and 79 MPa, respectively. The modulus of elasticity is 26,957 MPa and the Poisson’s ratio is 0.3. 

**Figure 5:**
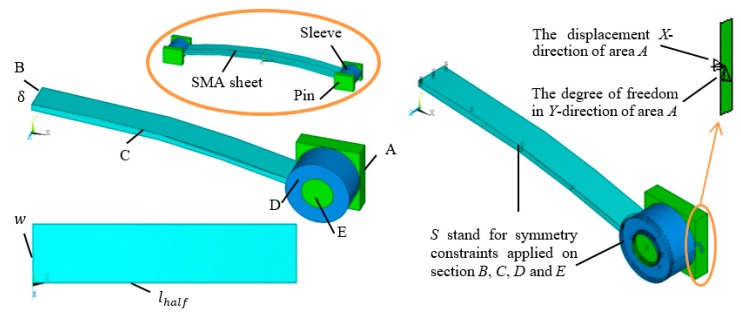
The C-shaped SMA model and the 1/4 part of the model for $l_{half}$ as a design variable used in optimization.

The designed constant force component is composed of a C-shaped superelastic SMA sheet, two sleeves, and two pins. The structure of the model is shown in Figure 5. The pins and sleeves were designed to simulate loading on the ends of the sheet. The sleeves were rigidly connected to the superelastic SMA sheet. The pin and sleeve were set to be a rigid body with an elastic modulus of 150,000MPa and Poisson’s ratio of 0.3. Connection between the sleeve and the pin was set as surface-to-surface contact. In simulation, a 1/4 model was applied due to the symmetry. Symmetry constraints were applied on sections *B*, *C*, *D,* and *E*, and the degree of freedom in the *Y*-direction of Area *A* was constrained and the displacement in the *X*-direction of Area *A* was applied, as shown in Figure 5. The reaction force in the *X*-direction of Area *A* was obtained during the numerical simulation. The force–displacement values during all applying displacements were analysed.

**Figure 6:**
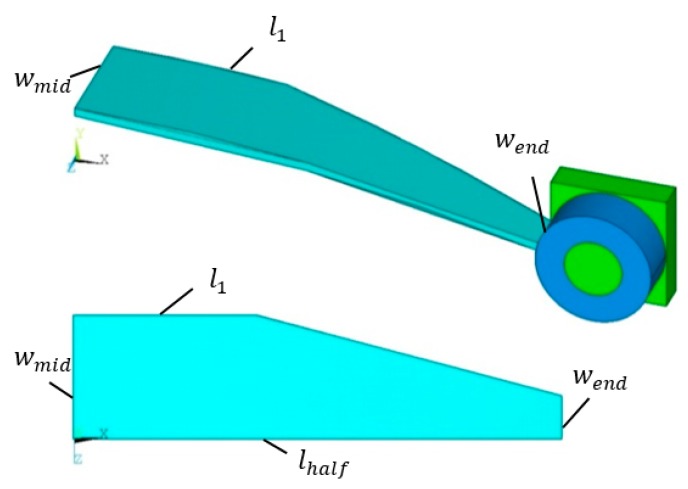
The model for the changed section width of sheet $l_{1}\;$ and $w_{end}$ as the design variable.

The equation for the initial C-shaped curve of the sheet is y(x) = $\frac{-1}{l_{half}^{2}}$x^2^ + 1, x ∈ [−$l_{half}$,$l_{half}$]. Different initial shapes of the sheet could be obtained by changing values of $l_{half}$. The optimization cases are as follows:

Case 1: The width, *w*, is set to be 1 mm, $l_{half}$ is defined as the design variable, and its lower and upper bounds are 4 and 15, as shown in Figure 5.
Case 2: The value of $l_{half}$ is set to be 11 mm—namely, the C-shaped curve equation of the SMA sheet is fixed as y = (−1/121) x^2^+ 1. The width at the middle of sheet *w_mid_* is set to be 2 mm, and *w_end_* and *l*_1_ are as the design variables. The section width of the sheet is changed linearly along its longitudinal direction, as shown in Figure 6.

### 2.3. The Experiment Verification for the Generalized Design Method 

**Figure 7:**
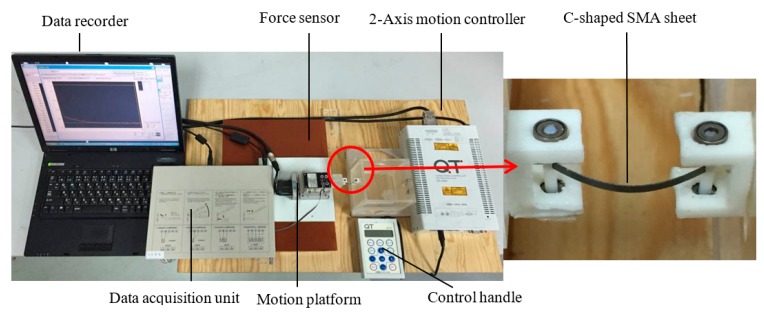
Experimental setup validating the generalized design method.

The design results of the generalized design method were validated by performing the experiment on real prototypes. The experimental test sample with 0.2 mm thickness were first cut out from the large SMA sheet by wire-electrode cutting, and then constrained in a custom-made mold for forming heat treatment. The experimental setup was constructed for measuring the actual force–displacement curve, as shown in Figure 7. The material, parameters, boundary, and loading condition used in the experiment are exactly the same as those described in the optimization simulation. The *X*-displacement load was applied on the ends of the sheet by the 2-Axis motion controller (CHUO SEIKI, QT-CM2), and the reaction forces at the ends of the sheet were measured by a force sensor (NMB, UT-500GR). As the C-shaped SMA sheet moved from *D* = 0 to *l −* 2 mm along the *X*-direction, reaction forces *F* were recorded in the computer by a data acquisition unit (KYOWA, PCD-300A).

## 3. Results

**Figure 8:**
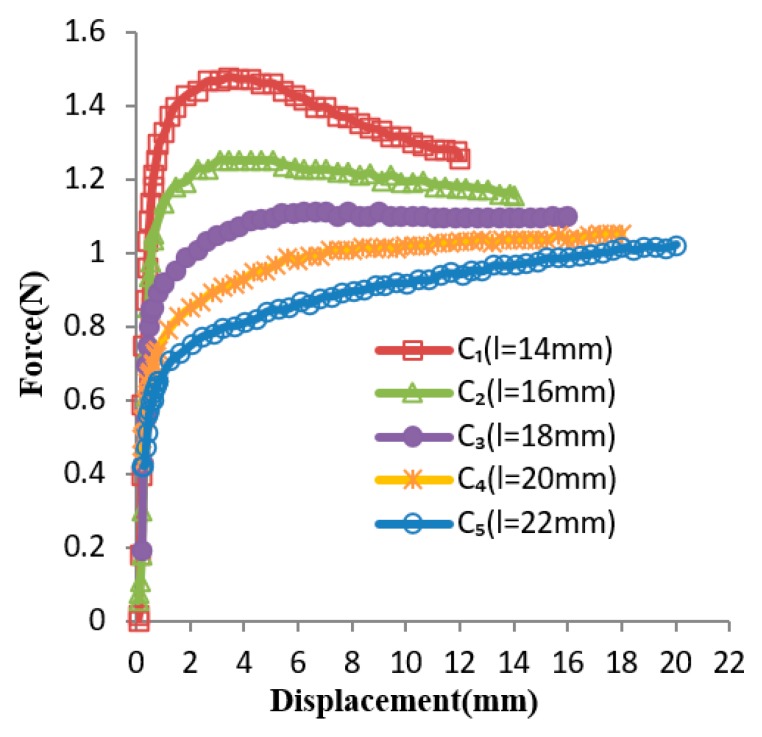
Force–displacement experimental curves, with $l_{half}$ as the design variable.

**Figure 9:**
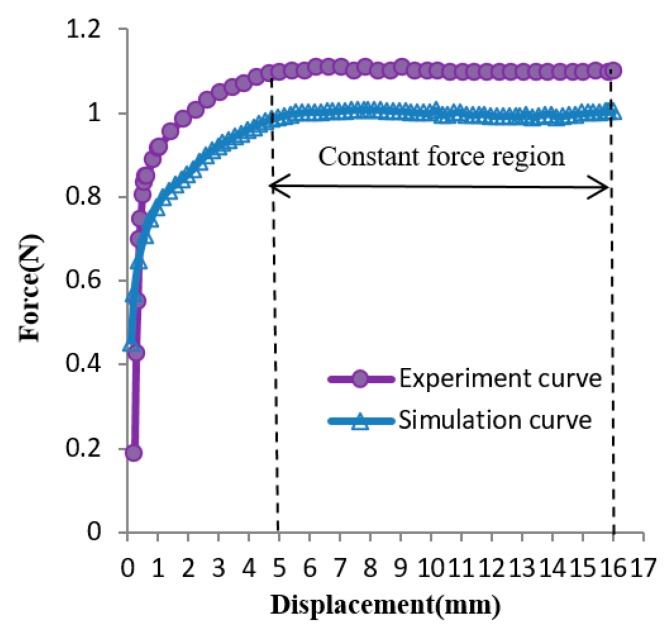
Comparison of the optimal simulated curve (at l = 18 mm) with its corresponding experimental curve.

Based on the generalized design method, the design solution obtained for Case 1 was *l* = 18 mm. The several experiment curves of the C-shaped sheet for Case 1 are shown in Figure 8. The curves *C*_1_, *C*_2_, *C*_3_, *C*_4_, and *C*_5_ are force–displacement curves of SMA sheets, while the variable *l* was set to be 14 mm, 16 mm, 18 mm, 20 mm, and 22 mm, respectively. It can be seen that Curve *C*_3_ (*l* = 18 mm) is the optimum, as its slope remained nearly constant within a certain range of displacement, which is coincident with the result obtained by the generalized design method. Figure 9 shows the comparison between the optimal design simulation curve and the experimental curve of the C-shaped sheet with *l* = 18 mm, and the experimental curve is roughly 1.11 N higher than its optimal design simulation curve in the constant force region. They all show good flatness between a displacement of 5 and 16 mm. 

**Figure 10:**
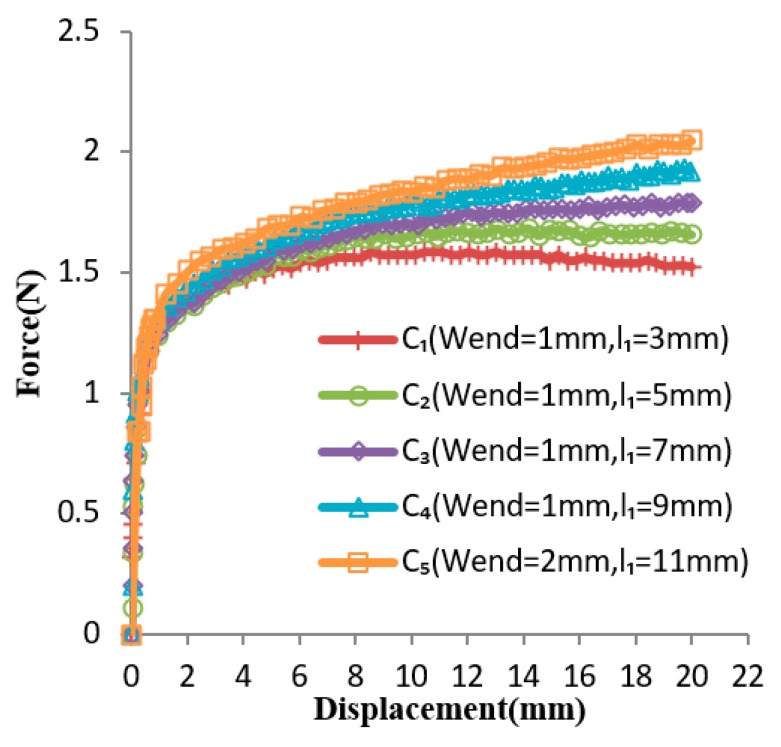
Several experimental curves at changing section widths of the sheet.

**Figure 11:**
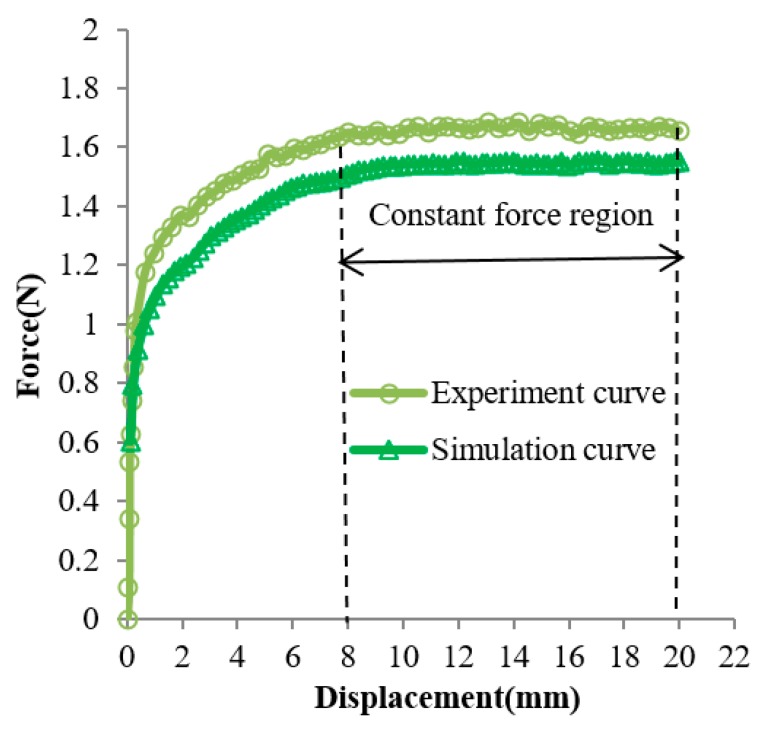
Comparison of optimal simulated curve $\left(\;w_{end}=1\;\mathrm{mm},l_{1}=5\;\mathrm{mm}\right)$ with its corresponding experimental curve at changing section widths.

Based on the generalized design method, the design solution obtained for Case 2 is *w_end_* = 1 and *l*_1_ = 5 mm. The several experiment curves of the C-shaped sheet for Case 2 are shown in Figure 10. The curves *C*_1_, *C*_2_, *C*_3_, *C*_4_, and *C*_5_ are force–displacement curves of SMA sheets with the variable *w_end_* = 1 and *l*_1_ of 3 mm, 5 mm, 7 mm, 9 mm, and *w_end_* = 2, *l*_1_ = 11 mm, respectively. It could be seen that curve *C*_2_ (*w_end_* = 1 and *l*_1_ = 5 mm) is the optimum, as its slope remained nearly constant within a certain range of displacement, which is coincident with the result obtained by the generalized design method. Figure 11 shows the comparison between the optimal design simulation curve and the experimental curve of the C-shaped sheet with *w_end_* = 1 and *l*_1_ = 5 mm, and the experimental curve is roughly 0.14 N higher than its design simulation curve in the constant force region. They all show good flatness between a displacement of 8 and 20 mm. 

## 4. Discussion

In Case 1, the optimal parameter value obtained through constant force by the experimental curve is coincident with the result obtained by the generalized design method. The experimental curve is roughly 1.11 N higher than its optimal design simulation curve in the constant force region. However, the trend of the experiment curve and optimal design simulation curve are consistent. They all show good flatness between a displacement of 5 and 16 mm. The constant force region has approximately 11 mm displacement. Obtaining the maximal displacements range that had a nearly constant force was defined as the optimization goal, and the objective function was formulated as Max (*D_m_* − *D_n_)*/*D_k_*. The constant force region was approximately 68.75% of the entire input displacement, and approximately 61.11% of the entire compressive strain. It can be seen from Figure 8 that the forces in the latter half of several curves gradually increase, and the forces in the latter half of several curves gradually decrease. Thus, there must have been a value of Parameter *l* to make the forces in the latter half of the curve tend to be constant. The large constant force region of a C-shaped SMA sheet could be obtained by simply varying its Parameter *l*. It is very useful for clamping devices in medical instruments which need a large displacements range or strain range that has a nearly constant force. A drawback of changing parameters *l* is that sometimes, the optimal value can be large, which limits its use and thus compromises the performance of these applications.

In Case 2, the optimal parameter value obtained by constant force in the experimental curve is coincident with the result obtained by the generalized design method. The experimental curve is roughly 0.14 N higher than its design simulation curve in the constant force region. Nevertheless, the shape of the experimental curve is similar to the optimal design simulation curve, and they both show good flatness between a displacement of 8 and 20 mm. The constant force region has approximately 12 mm displacement. The constant force region was approximately 60% of the entire input displacement, and approximately 54.55% of the entire compressive strain. It can be seen from Figure 10 that the forces in the latter half of several curves gradually decrease, and the forces in the latter half of several curves gradually increase. Thus, there must be a set of values for parameters *w_end_* and *l*_1_ to make the forces in the latter half of the curve tend to be constant. The large constant force region of a C-shaped SMA sheet could be obtained by linearly changing the width of the sheet along its longitudinal direction. When varying the length of the sheet sometimes has space limitations, it is highly advantageous to linearly change the width of the sheet along its longitudinal direction.

In view of the above two cases, the constant force property is not sensitive to modeling and manufacturing errors. The results of the experiments verified that it is feasible to adopt the generalized design method to generate a shape memory alloy constant force component. The constant force properties of a flat SMA component can only be obtained by the tensile deformation of the SMA. However, the constant force properties of a C-shaped SMA component can be obtained by its compression deformation, which may occupy small space and for which miniaturization is easy to achieve. It is highly advantageous for biomedical applications. Thus, the SMA constant force component may be embedded into hemostatic forceps to make the clamping force constant, which may prevent soft tissue injury from excessive pressure and slippage due to low pressure. To simplify the design, the generalized design method was limited so that the initial shape of SMA sheet was known. A generalized design method for the designing of an SMA constant force component-based arbitrary shape will be studied further later; at present, only the geometric parameters of SMA are optimized. Topology optimization for arbitrarily shaped SMA will continue to be studied in future work.

## 5. Conclusions

This paper proposes the generalized design method to generate a constant force component using a superelastic shape memory alloy. The designed results using the generalized design method and experimental results of the C-shaped shape memory alloy sheets verify feasibility and accuracy of the proposed generalized design method. It was demonstrated that a large constant force range can be obtained by varying the geometric dimensions of shape memory alloy component. More generalized method designs based on arbitrary shape will be carried out in the future.
